# Impact of plasmid architecture on stability and yEGFP3 reporter gene expression in a set of isomeric multicopy vectors in yeast

**DOI:** 10.1007/s00253-017-8558-0

**Published:** 2017-10-20

**Authors:** Ruben Hohnholz, Kim Julia Pohlmann, Tilman Achstetter

**Affiliations:** 1 City University of Applied Sciences Bremen, Neustadtswall 30, D-28199 Bremen, Germany; 2 Jacobs University Bremen, Campus Ring 1, D-28759 Bremen, Germany; 3 University of Bremen, Bibliothekstraße 1, D-28359 Bremen, Germany

**Keywords:** *Saccharomyces cerevisiae*, Episomal (multicopy) plasmids, Plasmid stability, Yeast, yEGFP3

## Abstract

Multicopy episomal plasmids in yeast, used whenever elevated levels of foreign or homologous gene expression are necessary, are known to be less stable compared to the endogenous 2-μm plasmid they are based on, at least without selective pressure. Considering that rich medium favors growth rate and, simultaneously, is less expensive than selective medium, enhancing stability in non-selective medium is extremely desirable. In this study, we changed the architecture of a multicopy model expression plasmid, creating six isoforms (same size, same DNA content but different positions and orientations of the expression block) and studied mitotic stability, copy number, as well as reporter yEGFP3 expression between isoforms. With one isoform being significantly more stable than the others and another one exhibiting elevated plasmid copy numbers in rich medium, we show that consideration of the arrangement of the plasmid elements might be crucial for productivity employing *Saccharomyces cerevisiae* as a host. We strongly believe that the ideal architecture has to be assessed for each case and assembly strategy has to begin by evaluating the stability of the vector backbone before insertion of the desired gene. For the plasmid set studied, yEGFP3 reporter production depends more on mitotic stability than on elevated plasmid copy numbers in a small number of cells retaining the plasmid under non-selective conditions.

## Introduction

Robustness of an industrial process employing recombinant organisms seems largely dictated by abiotic process parameters. Likewise important is the contribution of the producing organism itself. As a cell-based system, the host inevitably is subject to selection (and counterselection), disfavoring non-physiological changes of its genetics and metabolism. In order to overproduce proteins or low molecular weight metabolites, homologous or heterologous, almost always genetic information needs to be transformed into the host cell. The necessary sequences in most cases make part of a plasmid vector. The stability of such a vector in the recipient cell is of primordial importance for the robustness and productivity of the production system, instability might render the validation of an industrial process questionable or even impossible (Zhang et al. 1996; Delvigne and Goffin 2014; Gustavsson and Lee 2016).

Since a long time, the yeast *Saccharomyces cerevisiae* is a favorable host in industrial production, not only for food and alcoholic beverages, but also for biofuels, and, in particular, for high-value pharmaceuticals and vaccines (e.g., Ferrer-Miralles et al. 2009; Martinez et al. 2012; Nielsen 2013). Well-established genetics, high growth rates, a basic capacity for eukaryotic protein processing and secretion, and the “generally recognized as safe” (GRAS) status by the US Food and Drug Administration are just some of many beneficial traits of yeasts like *S. cerevisiae*. In addition, yeast has a somewhat outstanding position as it can use plasmids of various types, a feature rarely found in eukaryotic organisms (Gunge 1983; Caunt et al. 1988; Da Silva and Srikrishnan 2012). In the case of heterologous production, plasmids play an important role for easy genetic manipulation. DNA can be introduced by using YCp-type plasmids (yeast centromeric plasmids carrying a chromosomal origin of replication and additional centromeric sequences for segregational stability) or YIp-type plasmids (yeast integrative plasmids for genomic integration), both resulting in high stability. In both cases, expression levels are, however, generally low due to the presence of only one copy of the plasmid and thus of the gene of interest (Caunt et al. 1988; Romanos et al. 1992). In addition, integration might disrupt cellular functions and cause unfavorable regulatory control issues (Parker and DiBasio 1987). As an alternative, YEp-type plasmids (yeast episomal plasmids) do exist, carrying sequences of the yeast endogenous 2-μm plasmid. This 2-μm plasmid is found in most wild-type and laboratory *S. cerevisiae* strains (for a recent review on 2-μm structure and functions, see Chan et al. 2013). YEp-type plasmids are present in multiple copies and thus promise a benefit from a gene-dosage effect in terms of an increased productivity (Romanos et al. 1992; Da Silva and Srikrishnan 2012). The downside of using YEp-type vectors with an elevated copy number is a reduced stability. Instability can be either structural or segregational, the latter caused by uneven partitioning of plasmids during cell division (Caunt et al. 1988). Research often employs yeast as a model system in a multitude of applications. Here too, YEp-type plasmids are frequently used, though their stability seems of minor concern and is rarely questioned. Nevertheless, problems linked to selection and counterselection apply also on a small scale. Taking those problems in consideration, experimental results sometimes might ask for a second view.

Attempts have been made to increase stability and optimize productivity of YEp-type plasmids. Environmental approaches for enhancing stability include media formulation and fermentation conditions and have been discussed in detail (Zhang et al. 1996; Caunt et al. 1988). Using genetic approaches such as different yeast selectable markers and promoters, vector sets for optimized metabolic engineering have been constructed (e.g., Fang et al. 2011; Karim et al. 2013; Seresht et al. 2013; Gnügge et al. 2016; reviewed in Da Silva and Srikrishnan 2012). Complementation of chromosomal auxotrophic markers with a functional copy of the gene contained on the plasmid would allow a transformed strain to grow in selective minimal media (Sherman 1991). Likewise, a yeast-adapted gene carried on the plasmid could confer a resistance to antibiotics. Both systems are commonly used in small scale and result in high stability while maintaining a high copy number. In any case, both conditions are not feasible in large-scale production due to high costs and, in the latter case, an intensive downstream processing (Gustavsson and Lee 2016).

**Fig. 1:**
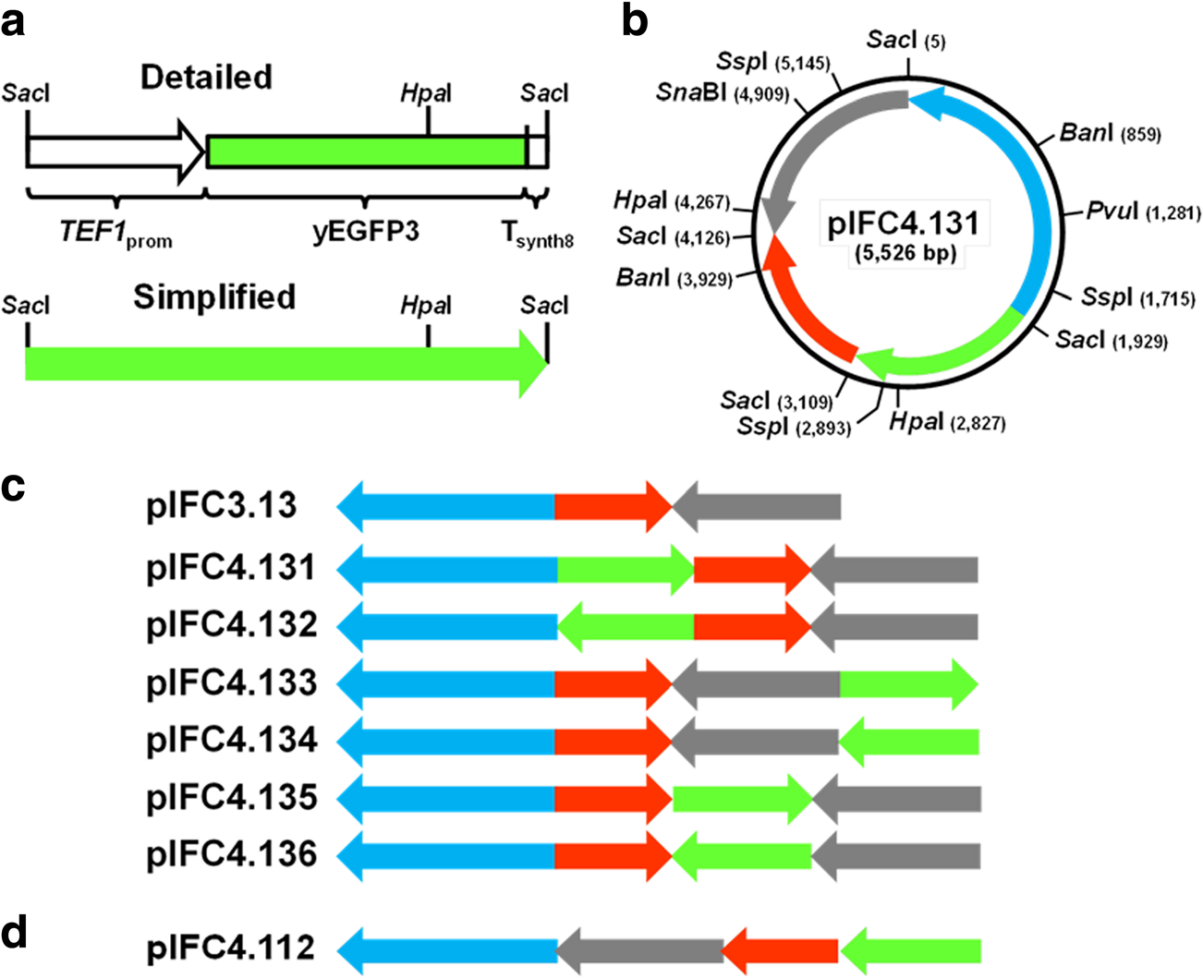
Isomeric forms of an *E. coli*-yeast shuttle vector (2 μm, *HIS3*) carrying a synthetic yEGFP3 expression block **a** yEGFP3 expression block with the *TEF1* promoter (*TEF1*_prom_ *)*, the yEGFP3 coding sequence, and the artificial transcription termination sequence T_synth8_ (Curran et al. 2015). The expression block is flanked by *Sac*I recognition sequences. , schematic drawing of the expression block, where the arrow marks the direction of transcription. **b** pIFC4.131 with the *Sac*I sites indicating fragments borders, as well as asymmetric sites which allow to distinguish fragment orientation. , bacterial fragment (1924 bp) with *oriV* and *bla*, where the arrow marks the direction of transcription of the *bla* gene; , yeast 2 μm fragment (1405 bp), where the arrow marks the direction of transcription of the *FLP* gene; , *S. cerevisiae HIS3* gene (1017 bp), where the arrow marks the direction of transcription; , yEGFP3 expression block. **c** pIFC4.13X plasmid family (schematic drawing); pIFC3.13 is 4.346 bp long, pIFC4.131–4.136 plasmids are 5526 bp long. **d** pIFC4.112, expression vector, derived from pIFC3.11 (Hohnholz et al. 2017)

In continuation to a previous study, expression model vectors were constructed based on a published YEp-type multicopy plasmid (plasmid isoform construct [pIFC]3.13; Hohnholz et al. 2017). A family of plasmids varying in their architecture was assembled by addition of a yEGFP3-based reporter gene in different positions and orientations. This enabled us to study the potential impact of plasmid-derived (heterologous) gene expression on plasmid loss, plasmid copy numbers (PCNs), and levels of reporter protein expression. GFP or its variants are frequently used in cell and molecular biology studies, also in *S. cerevisiae*. Fluorescent proteins are thought to be without major physiological consequences for the host cell even at high expression levels. Expression of our reporter is under control of the strong constitutive yeast *TEF1* promoter (Nacken et al. 1996; Sun et al. 2012; Lee et al. 2013; Peng et al. 2015), and transcription is terminated by the short synthetic T_synth8_ sequence (Curran et al. 2015) (Fig. 1a). To our knowledge, this is the first time that architectural rearrangements have been analyzed systematically to investigate stability and reporter protein expression in yeast. Unexpectedly and despite being isoforms, the arrangement of plasmid elements has an impact on mitotic stability, copy number, and protein expression in non-selective media. We demonstrate thus that it can be of significance to modify the arrangement of functional elements of an expression plasmid. Our data suggest that one isoform, pIFC4.134, represents a particularly favorable arrangement of the functional sequences, combining high stability and the highest reporter protein expression under non-selective conditions. The percentage of transformed cells in a culture seems more relevant to overall heterologous productivity than the contribution of a small number of cells carrying a high PCN.

## Materials and methods

A family of isomeric multicopy model expression plasmids of 5526 bp was created based on pIFC3.13, a particularly stable member of the aforementioned pIFC3.X series (Hohnholz et al. 2017). These plasmids carry an expression block with the yeast-adapted yEGFP3 serving as reporter protein.

### *Escherichia coli* and yeast strains

For cloning and DNA amplification, *E. coli* strain DH5α (Life Technologies, Darmstadt, Germany) was used employing standard molecular biology protocols. As we did not see a difference in transformation efficiency and segregational stability between the two common laboratory *S. cerevisiae* strains SY922 and BY4742 (Hohnholz et al. 2017), we focused in this study on the widely employed BY4742 (*MAT* ***α***, *his3Δ1*, *leu2Δ0*, *lys2Δ0*, *ura3Δ0;* Brachmann et al. 1998; Euroscarf collection, Frankfurt, Germany). *E. coli* and yeast transformation and propagation were done as described previously (Hohnholz et al. 2017).

### Assembly of the yEGFP3 expression block and plasmid construction

The yEGFP3 expression block (1180 bp; Fig. 1a) consists of a fragment of the strong constitutive yeast *TEF1* promoter, a yeast-adapted synthetic sequence encoding a mutated version of the *Aequorea victoria* GFP (S65G, S72A; Cormack et al. 1997), and the T_synth8_ synthetic transcription termination sequence (Curran et al. 2015). *TEF1* promoter sequences (YPR080W; 407 bp) were derived by PCR from yeast strain SY992 (Tomlin et al. 2001) with a *Sac*I site added to the 5′ end. yEGFP3 sequences (714 bp) were amplified from pUG34 (supplied by J. H. Hegemann, Heinrich-Heine-Universität, Düsseldorf, Germany) adding a TAG stop codon to their 3′ end. The T_synth8_ synthetic terminator (49 bp), preceded by an A, with 6 bp added to its 3′ end for a *Sac*I site, were assembled from a pair of oligonucleotides. Oligonucleotides used in the assembly are

RH007 5′-TATGAGCTCCCACACACCATAGCTTCAAAATG-3′,

RH008 5′-AATTCTTCACCTTTAGACATCTTAGATTAGATTGCTATGCTTTCTTTCTAATGAGC-3′,

RH009 5′-GCATAGCAATCTAATCTAAGATGTCTAAAGGTGAAGAATTATTCACTGGTG-3′, and RH010 5′-TATGAGCTCTTTGAAAGATGATACTCTTTATTCCTACATAAGTAAATGAGTTTATATATCTATTTGTACAATTCATCCATACCATGGGTAATACC-3′. The yEGFP3 expression block was inserted in pUC18 from where it was recovered as a *Sac*I-*Sac*I fragment.

### Cloning of the expression plasmids pIFC4.131–4.136 and yeast transformation

The *Sac*I fragment of the yEGFP3 expression block was cloned into pIFC3.13 (Hohnholz et al. 2017) partially digested with *Sac*I. The complete series of isomeric expression plasmids was recovered with the reporter expression block inserted in all three possible positions and, in each case, in both orientations (Fig. 1c). The yEGFP3 expression block was also inserted into pIFC3.11 in the same manner, recovering pIFC4.112. For the purpose of this work, we transformed yeast strain BY4742 with each member of the series (Fig. 1c) employing a published method (Gietz and Schiestl 2007).

### Plasmid loss studies

His^+^ transformants of each plasmid were cultivated for 16 h in selective SD_sup_ (fully synthethic media with dextrose as carbon source and with L-leucine, L-lysine, and uracil as supplements) and for three consecutive cycles of 24 h of cultivation (= 72 h) in SD _sup_ starting with a 1:1000 dilution or in YPDAU (YPD supplemented with adenine and uracil) starting with a 1:4000 dilution as described for previous plasmid loss studies of the pIFC3.X plasmid family unless otherwise stated (Hohnholz et al. 2017). Plasmid loss rates were calculated in the published manner (Hohnholz et al. 2017). As we expected segregational stability to drop for the expression vectors, we followed plasmid loss for a reduced time, i.e., 3 × 24 h = 72 h instead of 5 × 24 h = 120 h (in our case more than 30 generations).

### Plasmid copy numbers

DNA was prepared, and PCNs were assessed by qPCR with the PerfeCTa® SYBR® Green FastMix (Quanta, Beverly, MA, USA). Detection system and analysis software were the Mastercycler® RealPlex^2^ and *realplex* software version 2.2 (Eppendorf, Hamburg, Germany). A 113 bp fragment of the *HIS3* ORF (YOR202W) contained in the plasmid was amplified, likewise a 111 bp fragment of the chromosomal single copy gene *ENB1* (YOL158C) serving as internal standard (Hohnholz et al. 2017).

### Reporter gene expression analysis

His^+^ transformants (a mixture of 10 colonies randomly chosen) were recovered from a SD_sup_ agar plate and were grown overnight in liquid SD_sup_ at 30 °C or for 48 h in YPDAU as described for the plasmid loss studies. Cells were harvested, washed, and suspended in a 1:1 ratio of cells to potassium phosphate buffer (50 mM, pH 6.0) containing a protease inhibitor mix (Complete, Mini, EDTA-free, Roche Diagnostics, Mannheim, Germany). Glass beads (Ø 0.25–0.5 mm, Carl Roth, Karlsruhe, Germany) were added to the suspension. Cells were lysed by vortexing at V_max_ in 6 cycles of 2 min with intermittent 1 min cooling on ice. Extracts were recovered and glass beads were washed twice with 50 μL of ice cold buffer. Extracts and washings were pooled and centrifuged at 13,000 *x* g at 4 °C for 1 min. Supernatants were withdrawn and kept at −18 °C in aliquots for further use. Protein concentrations were assessed employing the Bradford protocol with bovine serum albumin as a standard.

Native polyacrylamide gel electrophoresis (PAGE) was carried out following the protocol of Bio-Rad (Bio-Rad Laboratories GmbH, Munich, Germany). Samples with the desired amount of protein were diluted in sample buffer (Bio-Rad) and were separated on commercial 4–20% polyacrylamide gradient gels (Bio-Rad) in running buffer (Tris-glycine; following the Bio-Rad protocol). Fluorescent bands were detected illuminating the gels with the Blue/Green LED Transilluminator (Nippon Genetics, Dueren, Germany) at 480–530 nm.

Relative fluorescence was determined using the Hitachi F-2500 Fluorescence Spectrophotometer (FL Solutions program; Hitachi High-Technologies Europe GmbH, Krefeld, Germany) with an excitation of 485 nm and an emission spectrum between 500 and 560 nm. For whole cell analyses, 5 OD_600_ units of cells were centrifuged, washed, and resuspended in 1.5 mL water. For protein extract analyses, 80 and 40 μg of total protein were diluted in 1.5 mL of water, respectively.

## Results

### Growth retardation and plasmid loss

Transformation efficiencies of the plasmid isoforms did not vary significantly, and, more importantly, with 1.1 × 10^6^ transformants per μg of plasmid DNA per 10^8^ cells, efficiency did not decrease compared to the parental pIFC3.13 and was in the range of published results (Gietz and Schiestl 2007; Mitrikeski 2015).

Results of growth behavior of His^+^ transformants and plasmid loss are summarized in Table 1. Growth was retarded by at least 10% when comparing the parental vector pIFC3.13 with the expression plasmids. Plasmid loss rate only doubled in the best of all cases (pIFC4.134, Table 1), for the other isomers loss rates increased by four to fivefold with less than 25% of His^+^ clones after about 30 generations of growth in YPDAU. Thus, the pIFC4.13X expression plasmids cause a significant slowdown in growth, and they exhibit a marked segregational instability in our plasmid loss protocol.

**Table 1:**
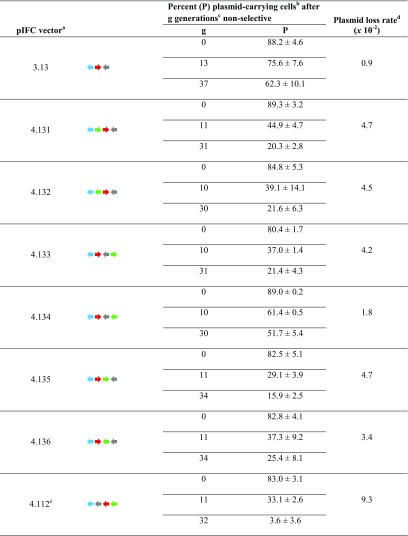
Mitotic stability, generation time, and loss rate of the pIFC4.13X plasmid family in BY4742

Three individual His^+^ transformants for each vector were inoculated into SD_sup_. OD_600_ was measured and 100 μl containing theoretically 150 cells were plated out immediately on YPDAU plates in triplicates. The colonies were scored for plasmid retention by replica plating onto selective agar. The mean and standard deviation of the percent (P) of plasmid-carrying cells in the inoculum was assessed. Standard deviation was calculated by standard methods and reflects the variation of the three individual His^+^ clones

^a^Vector schemes: see Material and Methods and Fig. 1

^b^P was calculated by analyzing clones from replica plating. On average, 779 cfu for each particular time and transformant were analyzed, but at least 311 cfu

^c^The doubling times were assessed as previously published (Hohnholz et al. 2017)

^d^Plasmid loss rates were assessed as previously published (Hohnholz et al. 2017). ^e^pIFC4.112 is derived from pIFC3.11 (Hohnholz et al. 2017)

**Fig. 2:**
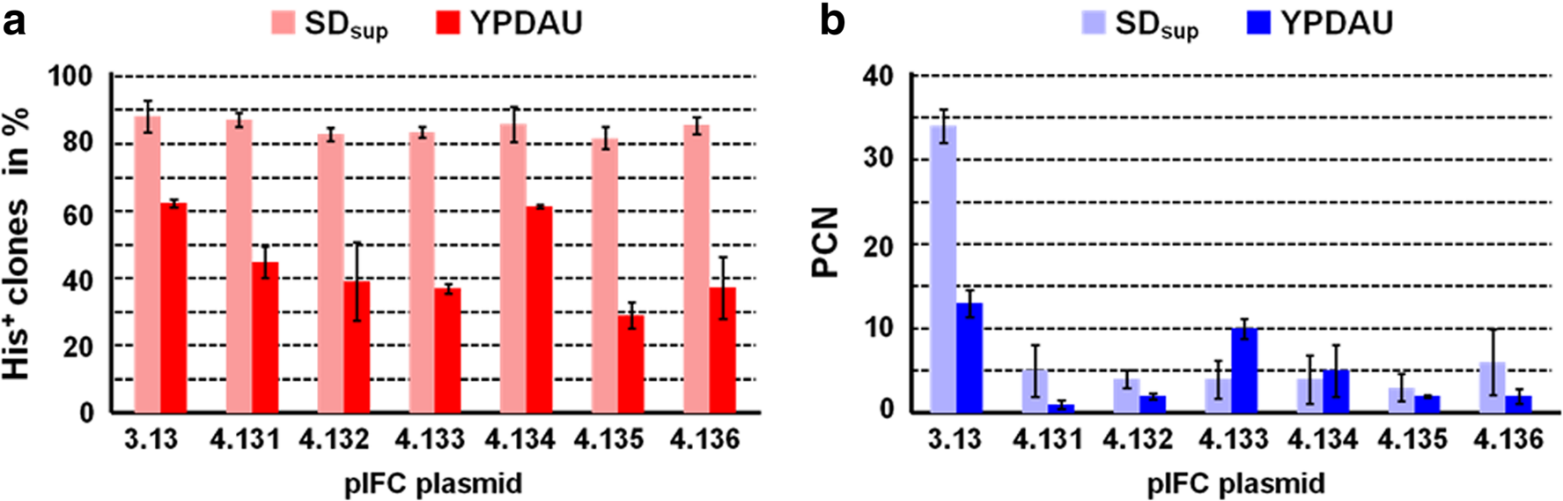
Copy number of model expression plasmids of the pIFC family His^+^ transformants of strain BY4742 were pregrown in SD_sup_ overnight (t0), corresponding to about 2–3 generations. Cells were then transferred to fresh SD_sup_ or YPDAU and were then cultivated for an additional 48 h with a change of media after 24 h, corresponding to additional 7–8 generations in SD_sup_ and 10–11 generations in YPDAU according to the plasmid loss protocol. DNA was prepared, and PCNs were determined as described in Materials and Methods. Standard deviations for the percentage of His^+^ clones (*n* = 3) were calculated as described in Table 1. PCNs shown are the mean results of three His^+^ clones with two biological and two technical replicates (± standard deviation). **a** His^+^ cells (in %) as determined by replica plating on selective media after preculturing in SD_sup_ overnight and subsequent propagation for 48 h in SD_sup_ or in YPDAU; **b** PCN after cultivation overnight in SD_sup_ (t0) and subsequent propagation for 48 h in YPDAU

As mentioned above, environmental factors affect plasmid stability. Media composition is one of those factors. In selective media, segregation stability of our isoforms is high and does not differ between the plasmids (81.5–86.7% His^+^ cells after 48 h in SD_sup_; Fig. 2a).

For expression plasmid pIFC4.112 (Fig. 1d), derived from a different backbone (pIFC3.11; Hohnholz et al. 2017), His^+^ clones made up less than 5% of the population after 32 generations in YPDAU.

### PCNs

In rich media, PCNs generally were lower than in SD_sup_ except for clones carrying pIFC4.133 (Fig. 2b). PCNs of the latter were more than two times higher in rich media compared to the average PCN of 2.4 (± 1.5) of the other five isoforms. PCNs did not vary to a larger extend between transformants carrying one of the pIFC4.13X vectors when the cells were propagated in selective conditions. With an average of 4.3 (± 1.0), PCNs in cells grown in SD_sup_ were, however, more than seven times lower when compared to the parental pIFC3.13 (Fig. 2b).

We also reasoned that only His^+^ clones carry the respective plasmid, thus we recalculated the PCNs for the fraction of transformed cells in the population, e.g., if PCN was determined to be 10 (per haploid genome), but only 25% of cells were His^+^, those cells carried indeed 40 plasmids and 75% no plasmid (i.e., had lost the plasmid). This calculation led to a three-fold increase in PCN in plasmid-carrying cells, particularly pronounced in cells with the mitotically unstable plasmids pIFC4.132-33 and 35-36 (data not shown).

### Reporter expression analyses

**Fig. 3:**
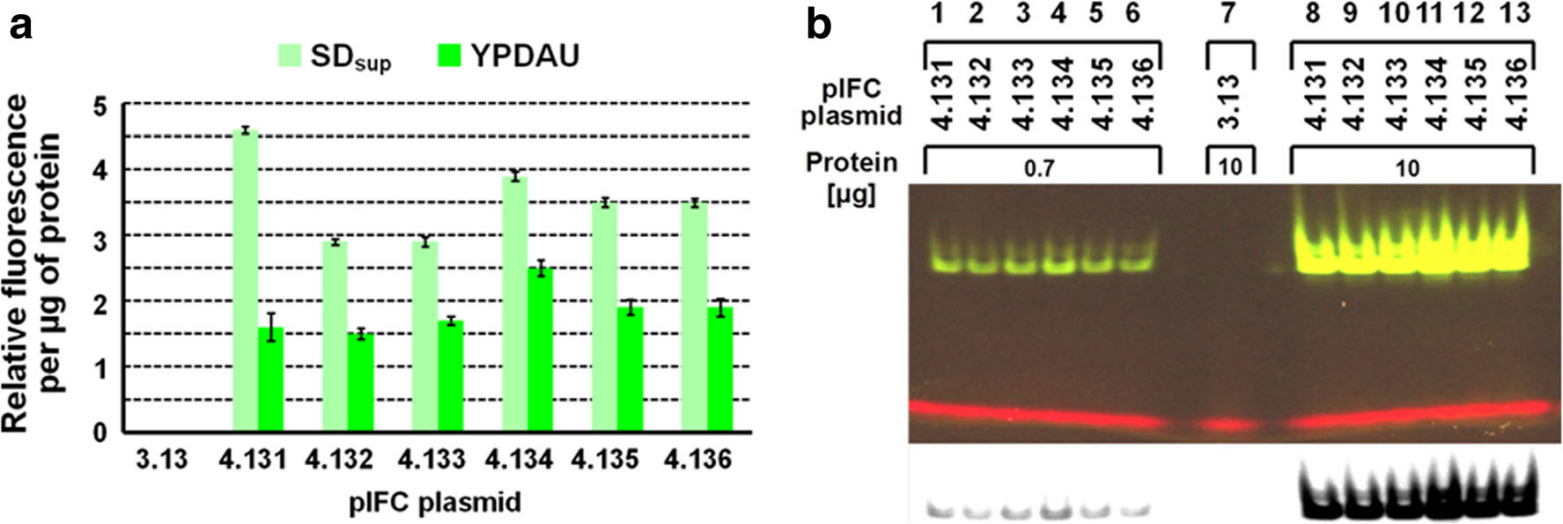
yEGFP3 produced from pIFC plasmids His^+^ transformants of strain BY4742 were grown as described in legend to Fig. 2; extracts were prepared, and their protein content was determined as described. Extracts from yeast clones with pIFC3.13 (no expression block) served as negative control. Extracts (**a**) were analyzed for their relative fluorescence as described, the mean of two technical replicates (± SD) is shown. Extracts from cells grown in YPDAU were separated by native PAGE (**b**) as described. Extracts from yeast clones with pIFC3.13 (no expression block) served as negative control (lane 7). Gels were exposed to LED (480–530 nm) as described. Pictures were taken (B, upper panel, shown is a picture which was enhanced (Microsoft Office PowerPoint 2007) for brightness and contrast equally across the entire image) and were converted to their negative form (presented as a black and white image for increased visual sensitivity). The camera was a Pentax MX-1 (Ricoh Imaging, Hamburg, Germany) and the image processing software used (B, lower panel) was ImageJ (NIH, Bethesda, MD, USA)

Cell-free extracts were analyzed for their relative fluorescence and thus for their content in yEGFP3 (Fig. 3a, b). The signal obtained is specific for yEGFP3 production as extracts from clones carrying the parental vector pIFC3.13 did not exhibit any detectable fluorescence. Among the six isoforms, pIFC4.134 allowed the highest productivity after 48 h in YPDAU with a 67% more intense fluorescence signal compared to pIFC4.132. When intact cells were analyzed, the results mirrored those of the extracts (data not shown). Expression levels for cells grown in SD_sup_ did vary by less than two fold comparing the six isomers. The same is true for cells grown in YPDAU, though their expression levels were generally lower (Fig. 3a). This analysis revealed that pIFC4.131 lead to an increased productivity in selective conditions by around 59% compared to pIFC4.132 and pIFC4.133 which showed the weakest fluorescence in the protein extracts (Fig. 3a).

Native PAGE indicated the presence of fluorescent molecules in those extracts at very low amounts of protein with pIFC4.134 giving a somewhat stronger signal than the rest (Fig. 3b, lanes 4, 11). Even at very high amounts of proteins, extracts of pIFC3.13 did not reveal any fluorescence (Fig. 3b, lane 7), confirming that fluorescence signals are linked to the presence of the expression plasmids. In PAGE, the fluorescent signal of yEGFP3 appeared to be derived from a doublet of bands, not always clearly visible (Fig. 3b). This phenomenon has been described recently where solvent molecules get “trapped” inside the yEGFP3, producing isoforms of the protein (Glukhova et al. 2017). In Coomassie-stained gels, likewise, a doublet of bands can be detected in those extracts (not shown).

## Discussion

Having observed that the arrangement of the functional segments of a simple multicopy yeast-*E. coli* shuttle plasmid of the YEp-type can have a significant effect on the segregational stability and PCN (Hohnholz et al. 2017), we wanted to go further in investigating the behavior of a set of isomeric expression plasmids.

In a previous study, the parental plasmid pIFC3.13 (Fig. 1) used here was found to exhibit a high segregational stability in strain BY4742 (43.6% in contrast to 4.6% of His^+^ cells carrying the isomeric pIFC3.21 in conditions resembling industrial cultivation, i.e., more than 60 generations in non-selective conditions) (Hohnholz et al. 2017). Choosing pIFC3.13 as a mitotically stable “backbone” for assembly of a model expression vector ensured the gain of comparable results for the plasmid isoforms harboring a reporter yEGFP3 expression block (Fig. 1a) as segregational stability was expected to drop, both due to an increased size and reporter gene expression (Parker and DiBasio 1987). In contrasts to pIFC3.13, segregational stability of pIFC3.11 was particularly low in the study mentioned (14.5% His^+^ cells after more than 60 generations). Insertion of the same yEGFP3 expression block (with pIFC4.112 a single form got cloned, Fig. 1d) produced another plasmid isoform to the pIFC4.13X family. BY4742 His^+^ transformants carrying pIFC4.112 had comparable growth rates but we observed a plasmid loss rate twice as high as the most unstable pIFC4.13X isoform (3.6% His^+^ cells after 32 generations; Table 1), hence insertion of the expression block further aggravated the segregational instability.

All members of the set (and also pIFC4.112) caused a noticeable growth retardation in non-selective conditions in *S. cerevisiae* (Table 1). Likewise, an increased plasmid loss could be observed. Nevertheless, one plasmid, i.e., pIFC4.134 (; see legend to Fig. 1 for explanation of the arrangement of the plasmid elements) performed significantly better than the other five. Plasmid loss rate was 2.6 times lower when compared to pIFC4.135 () and still 1.8 times lower compared to the second most stable plasmid pIFC4.136 () of the pIFC4.13X series after 30 or more generations.

For all six plasmids of the set, PCNs did not vary in SD_sup_; they dropped, however, markedly in both selective and non-selective conditions when compared to the parental pIFC3.13 (Fig. 2b). A small increase in size (of 27%) seems an unlikely explanation for the reduction as well as the observed growth retardation following the argument of Coppella and Dhurjati (1989). Therefore, the lower PCN must be the result of the addition of the yEGFP3 expression block to the plasmids. Its expression and subsequent reporter protein production seem to cause an adverse effect on the host favoring cells with a low PCN. A similar drop in copy numbers in His^+^ cells grown YPDAU in comparison to SD_sup_ has been observed for the set of isomeric basic pIFC3.X plasmids (Hohnholz et al. 2017).

Using rich media in large-scale production is the only economical option. However, in research and pilot-scale fermentation processes with *S. cerevisiae* as a host, employing selective media might be preferred. Reduced growth rates in those (data not shown) are balanced out by a high mitotic stability that in our case showed no differences between the isoforms (Fig. 2a).

Fluorescent yEGFP3 was chosen as a quantifiable reporter (of small size) for pIFC4.13X plasmid performance. In selective conditions, pIFC4.131 allowed for the highest reporter production in the plasmid family though differences between maximum and minimum production do in general not exceed 67% in YPDAU and 59% in SD_sup_ (Fig. 3a). Systematically, relative specific fluorescence derived from the pIFC4.13X plasmid family in cell-free extracts is two to threefold lower when transformants were propagated in YPDAU compared to those grown in SD_sup_ (Fig. 3a). Here, highest reporter production was with pIFC4.134 (Fig. 3a, b). The decrease in reporter production from cells grown in SD_sup_ to those grown in YPDAU seems to follow the drop in PCN with the exception of cells carrying pIFC4.133 when grown in non-selective conditions. Despite its relatively high copy number, cells with pIFC4.133 do not produce significantly more of the reporter in rich media pointing to limiting factors beyond the gene-dosage effect confirming published observations (Lee et al. 2015). pIFC4.134 seems to present a special case as fluorescence in extracts drops as for the other members of the set comparing SD_sup_ and YPDAU grown cells whereas its PCN increases. Furthermore, this plasmid stands out for its segregational stability in non-selective conditions (Table 1).

Overall, for the pIFC4.13X plasmid family, copy number and productivity seem to be linked to a certain degree. An average, PCN masks a potential uneven distribution in a population of cells (Caunt et al. 1988; Nevoigt et al. 2006) where a small fraction of transformants with particularly high PCNs cannot reach the same productivity as a larger number of plasmid-carrying cells with a lower PCN as they risk to run into limitations from metabolic resource shortage in protein production. We reason that to some extent, productivity and PCN are proportionally connected, however the number of transformed cells in the population seems to be the determinant for productivity rather than to their PCN as can be seen in the case of pIFC4.134 (Figs. 2 and 3). High-level production of any protein might have a toxic effect on the host cell. Fluorescent proteins are widely employed in this kind of study (e.g., Soboleski et al. 2005). Nevertheless, conflicting data exist indicating deleterious effects of fluorescent protein expression causing critical levels of hydrogen peroxide production (Ansari et al. 2016; Ganini et al. 2017) or even potential protective effects (Bou-Abdallah et al. 2006).

For the performance of pIFC4.134 showing the highest stability and reporter protein production, no offhand explanations can be given. Position and orientation of the expression block seem to play a role, particularly when comparing pIFC4.134 () to pIFC4.133 () where the turn-around of the expression block enhances both stability and productivity. Thus, the architecture of the isoforms seems to influence the performance of the reporter gene, which in turn influences stability possibly through accumulation of the yEGFP3 or transcription-transcription/replication interferences with neighboring sequences. To what extent chromatin structure or functionality of transcriptional terminators potentially causing pervasive transcription contribute to performance of the isoforms remains speculative (Marczynski and Jaehning 1985; Murray and Cesareni 1986).

Any high-level protein production will cause a metabolic burden to its respective host though this will not always become apparent in short time, small-scale experiments. It seems reasonable to assume that a change of the protein to be expressed will unpredictably influence PCNs and plasmid loss rates, e.g., in response to a potential specific toxicity of the gene product or an excessive demand of cellular resources. Thus, inevitably, assembly and optimization of any high-performance expression plasmid represent a case by case assessment.

In our opinion, a universal expression vector fulfilling all the requirements of a versatile research tool, easy to handle in terms of cloning and transformation and, at the same time, of a highly specific element for an industrial production system might not exist. Current molecular approaches consider promoters and terminators, selection markers, or host strains as adjusting screws to enhance the stability and productivity of an expression vector. The current work however demonstrates that plasmid stability, not PCN, dictates yEGFP3 productivity in yeast. The former in turn is influenced by the architecture of the expression plasmid. Having arbitrarily chosen the arrangement of elements on an expression plasmid and encountering high instability, and low copy numbers resulting in a low productivity, reconstructing the expression block or moving to an entirely different system might be more cumbersome than changing the architecture of the plasmid (including the backbone which might already be unfavorable).

In conclusion, changing the arrangement of the plasmid elements can boost stability and, as a consequence, productivity, carefully choosing both the backbone and the position and orientation of the expression block.
